# Body mass index trajectories and prostate cancer risk: Results from the EPICAP study

**DOI:** 10.1002/cam4.3241

**Published:** 2020-07-08

**Authors:** Céline Lavalette, Emilie Cordina Duverger, Fanny Artaud, Xavier Rébillard, Pierre‐Jean Lamy, Brigitte Trétarre, Sylvie Cénée, Florence Menegaux

**Affiliations:** ^1^ Université Paris‐Saclay UVSQ Inserm CESP Villejuif France; ^2^ Service Urologie Clinique Beau Soleil Montpellier France; ^3^ Labosud Institut médical d’Analyse Génomique‐Imagenome Montpellier France; ^4^ Registre des tumeurs de l'Hérault Montpellier France

**Keywords:** body mass index, obesity, prostate cancer, trajectory, weight gain

## Abstract

Elevated body mass index (BMI) has been inconsistently associated with prostate cancer occurrence but it has been suggested that life course adulthood obesity may be associated with an increased risk of prostate cancer. However, few studies have investigated lifetime BMI and prostate cancer risk. We analyzed life course BMI trajectories on prostate cancer risk based on data from the Epidemiological study of Prostate Cancer (EPICAP). We included in our analyses 781 incident prostate cancer cases and 829 controls frequency matched by age. Participants were asked about their weight every decade from age 20 to two years before reference date. BMI trajectories were determined using group‐based trajectory modeling to identify groups of men with similar patterns of BMI changes. We identified five BMI trajectories groups. Men with a normal BMI at age 20 developing overweight or obesity during adulthood were at increased risk of aggressive prostate cancer compared to men who maintained a normal BMI. Our results suggest that BMI trajectories resulting in overweight or obesity during adulthood are associated with an increased risk of aggressive prostate cancer, particularly in never smokers, emphasizing the importance of maintaining a healthy BMI throughout adulthood.

## Graphical abstract

The influence of body weight during lifetime on PCa risk remains largely ununderstood and has been poorly investigated. Our findings suggest that elevated BMI during adulthood resulting in overweight or obesity is associated with an increased risk of aggressive PCa, emphasizing the importance of maintaining a healthy BMI throughout adulthood.

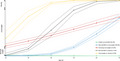

## 1 INTRODUCTION

The prevalence of obesity in the French population has increased in recent decades from 8.5% in 1997 to 15% in 2012.^1^ In 2016, 41% and 16% of French men were overweight or obese, respectively, resulting in major public health issues.^2^ According to the World Cancer Research Fund (WCRF) and the American Institute for Cancer Research (AICR), obesity during adulthood has been associated with several cancers, including breast in post‐menopausal women, endometrium, kidney, liver, colorectum, and pancreas.^3^ However, the link between obesity, assessed by body mass index (BMI), and prostate cancer is still under debate, with inconsistent results across studies, especially regarding overall or nonaggressive prostate cancer.^4^ A recent systematic review and meta‐analysis showed that an elevated BMI was associated with an increased risk of aggressive prostate cancer,^5^ also observed in recent studies.^6^, ^7^ Interestingly, a dose‐response meta‐analysis of nine observational studies that were able to examine adult weight gain and prostate cancer risk did not show any association with overall prostate cancer, but showed a positive association with high‐risk prostate cancer and prostate cancer mortality, respectively, based on only five and three studies.^8^

Considering the long period of induction of carcinogenesis, the influence of weight changes or obesity on cancer risk may vary throughout adult life. Inconsistent results have been reported between weight changes during adulthood and prostate cancer risk.^8^, ^9^ The majority of studies investing the association between obesity and prostate cancer either used static measures of BMI at one time‐point or BMI changes between only two time‐points that might be insufficient to capture the appropriate etiologic window of prostate cancer. In the meanwhile, BMI trajectories have been suggesting to better predict health outcomes than static measures.^10^ To date, only four studies were able to investigate body shapes or BMI trajectories over adulthood on prostate cancer risk,^11^, ^12^, ^13^, ^14^ suggesting a higher risk of overall and aggressive prostate cancer for men progressing from normal weight to overweight or obesity, compared to men with stable normal BMI.

In that context, we aimed to study life course BMI trajectories in relation to prostate cancer risk, using data from the Epidemiological study of Prostate Cancer (EPICAP).

## 2 MATERIAL AND METHODS

### 2.1 Study population

EPICAP is a population‐based case‐control study carried out in the *département* of Hérault, a well delimited geographic area in the South of France. Details of the EPICAP objectives and study design have been previously described.^7^, ^15^ In brief, cases were men newly diagnosed for prostate cancer in 2012‐2013, aged under 75 and living in the *département* of Hérault at time of diagnosis. Controls were men randomly selected from the general population, frequency‐matched to the cases by 5‐year age group, living in the same *département* as the cases and without history of prostate cancer at the time of inclusion. Quotas by socioeconomic status (SES) were set a priori to control for potential selection bias arising from differential participation rates across SES categories. These quotas were computed using census data available in the *département* of Hérault, in order to obtain a distribution by SES among controls similar to the SES distribution of the general male population of Hérault, conditionally to age.

Overall, 819 incident prostate cancer cases and 879 population‐based controls were recruited with a participation rate of 75% and 79%, respectively. All participants included in the study provided a written consent.

### 2.2 Data collection

Cases and controls provided information about socio‐demographic characteristics, occupational and residential history, lifestyle and leisure activities, personal and family medical history and anthropometric factors using a face‐to‐face standardized computerized questionnaire (CAPI—Computer Assisted Personal Interview) realized by research clinical nurses.

For cases, clinical data such as Gleason scores, Prostate Specific Antigen (PSA) levels, and tumor stage at diagnosis were extracted from patient's medical records and validated by the Hérault Cancer Registry.^16^

#### 2.2.1 BMI and history of BMI

Cases and controls were asked about their height at 20 years old and their weight every decade from age 20 to two years before reference date (age at diagnosis for cases and age at interview for controls).

BMI at each decade was calculated as self‐reported weight at each decade in kilograms divided by the square of the height in meters at age 20. We categorized each BMI according to the World Health Organization (WHO) definition into three classes: under‐weight and normal weight (BMI < 25 kg/m^2^), overweight (BMI: 25‐29.9 kg/m^2^), and obese (BMI ≥ 30 kg/m^2^).

#### 2.2.2 Covariates

Several covariates that may interfere with BMI over lifetime have been assessed: educational level, smoking status, alcohol consumption, physical activity, personal history of cardiovascular diseases or diabetes.

Smoking status was categorized as never, former, or current smoker. Alcohol consumption was assessed using the CAGE questionnaire^17^ in men who answered “Yes” to the question “Have you drink more than once a month during one year?”. Alcohol drinking has been categorized into three categories: never drinker (less than once a month during 1 year), low drinker (at least once a month during 1 year and zero or one positive answer to the CAGE questionnaire), and heavy drinker (at least once a month during 1 year and two or more positive answers to the CAGE questionnaire). Physical activity was assessed using Metabolic Equivalent Task (MET ‐ h/wk/yr)^18^ for each activity that has been practiced at least one hour per week during 1 year. We classified this variable into quartiles calculated in the control population: Q1 (<6.25 MET‐h/wk/yr), Q2 (6.25‐13.0 MET‐h/wk/yr), Q3 (13.0‐24.15 MET‐h/wk/yr), Q4 (≥24.15 MET‐h/wk/yr).

### 2.3 Statistical analysis

Analyses were performed in men who had no more than one missing data on body mass index history resulting in a final population of 1610 men (781 cases and 829 controls). On average, men reported 5.0 BMI over lifetime (minimum: 2; maximum: 6).

BMI trajectories were defined using group‐based trajectory models to identify and define groups of men with similar patterns of BMI change over lifetime, taking into account BMI as a categorical variable.^19^ We tested models including three to five trajectories using linear, quadratic and cubic polynomials. The optimal model (number of trajectories) was determined based on three criteria: Bayesian Information Criterion (BIC) (lower absolute values correspond to better fit); posterior probabilities of group assignment (the likelihood that an individual belongs to a given trajectory; all trajectories should have a mean posterior probability ≥ 0.70 and a minimum of 1% of participants per trajectory).^20^, ^21^ This method was implemented with PROC TRAJ SAS 9.4.

Unconditional logistic regression models were used to estimate odds ratios (ORs) and their 95% Confidence Interval (CI) to assess the role of BMI trajectories in prostate cancer risk. Analyses were systematically adjusted for age (5‐year groups), family history of prostate cancer in first‐degree relatives and ethnic origin (Caucasians, others).

Analyses were adjusted for potential confounding factors such as educational level, smoking status, alcohol consumption or physical activity. Since smoking is a risk factor for several cancer^22^ and may also interfere with weight changes,^23^, ^24^ we stratified our analyses on smoking status.

We conducted separate analyses by prostate cancer aggressiveness according to Gleason score at diagnosis (low or intermediate aggressiveness: Gleason score < 7 or Gleason score = 7 including subjects for whom the two most commonly represented grades in the tumor are 3 + 4, high aggressiveness: Gleason score ≥ 8 or Gleason score = 7 including subjects for whom the two grades are 4 + 3)).

We also performed sensitivity analysis in men with complete data regarding history of BMI (745 cases and 788 controls).

## 3 RESULTS

The characteristics of the EPICAP final population of analysis are presented in Table 1. Among prostate cancer cases, 77.1% were categorized as low or intermediate aggressive prostate cancer and 22.9% as aggressive prostate cancer. Age in 5‐year groups was similarly distributed between cases and controls. Considering sociodemographic and lifestyle characteristics, cases and controls were similar in terms of educational level, alcohol consumption and physical activity level. However, controls were more current smokers than cases. Personal history of cardiovascular diseases (myocardial infarction, angina pectoris, stroke) and diabetes history was also similarly distributed between cases and controls. Among our population of analysis, 460 men were classified as normal weight (29.1%), 759 (48.0%) as overweight, and 361 (22.8%) as obese at the reference date, similarly distributed between cases and controls. As expected, family history of prostate cancer in first‐degree relatives was more frequent in cases than in controls.

**TABLE 1:** Characteristics of the EPICAP final population of analysis, with at most one missing data on body mass index

	Cases n = 781 (%)	Controls n = 829 (%)	*P*‐value^a^
Gleason score			
<7	323 (42.0)	—	
7 (only 3 + 4)	270 (35.1)	—	
≥7 (including 4 + 3)	176 (22.9)	—	
Age (y)			.20
<55	47 (6.0)	57 (6.9)	
55‐59	96 (12.3)	95 (11.5)	
60‐64	205 (26.2)	191 (23.0)	
65‐69	263 (33.7)	268 (32.3)	
≥70	170 (21.8)	218 (26.3)	
Ethnic origin			.43
Caucasian	758 (97.1)	810 (97.7)	
Others	23 (2.9)	19 (2.3)	
Family history of prostate cancer in first‐degree relatives			<.0001
No	528 (75.9)	685 (90.3)	
Yes	168 (24.1)	74 (9.7)	
Body mass index at reference date^b^			.76
<25	221 (28.6)	239 (29.7)	
25‐29	380 (49.1)	379 (47.0)	
≥30	173 (22.3)	188 (23.3)	
Educational level			.29
Less than high school	422 (54.0)	476 (57.4)	
High school graduate	106 (13.6)	106 (12.8)	
College graduate	253 (32.4)	247 (29.8)	
Smoking status			.03
Never smoker	229 (29.4)	232 (28.0)	
Former smoker	441 (56.5)	442 (53.3)	
Current smoker	110 (14.1)	155 (18.7)	
Alcohol drinking^c^			.14
Never	67 (8.6)	75 (9.0)	
Low drinker	544 (69.6)	542 (65.4)	
Heavy drinker	170 (21.8)	212 (25.6)	
Physical activity			.24
No^d^	173 (22.3)	160 (19.4)	
<6.25 MET^e^‐h/wk/yr	147 (19.0)	165 (20.0)	
6.25‐13.0 MET‐h/wk/yr	132 (17.0)	163 (19.8)	
13.0‐24.15 MET‐h/wk/yr	143 (18.4)	167 (20.2)	
≥24.15 MET‐h/wk/yr	181 (23.3)	170 (20.6)	
Personal history of cardiovascular disease^f^			.95
No	699 (89.7)	737 (89.6)	
Yes	80 (10.3)	86 (10.4)	
Diabetes history			.87
No	677 (86.8)	712 (86.3)	
Yes	103 (13.2)	113 (13.7)	
Treated	91 (89.2)	103 (91.2)	

^a^ Adjusted for age (excepted for age).

^b^ Reference date: age at diagnosis for cases and age at interview for controls.

^c^ Never: Less than once a month during 1 y; Low drinker: at least once a month during 1 y and zero or one positive answer to the CAGE questionnaire; Heavy drinker: at least once a month during 1 y and two or more positive answer to the CAGE questionnaire.

^d^ No: Less than 1 hr/wk during at least 1 y.

^e^ MET: Metabolic Equivalent Task.

^f^ Myocardial infarction, angina pectoris, stroke.

**FIGURE 1:**
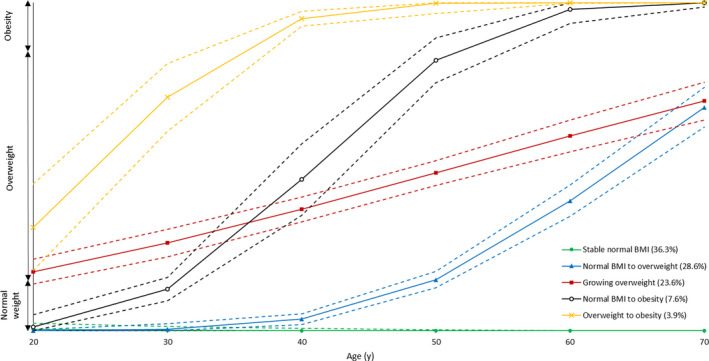
Body mass index (BMI) trajectories in the EPICAP study in men with at most one missing data on BMI

We identified five distinct trajectories (linear polynomials) of categorical BMI between age 20 and two years before reference date (Figure 1). In our population, 584 men (36.3%) maintained a normal BMI, 460 (28.6%) had normal BMI and became overweight, 381 (23.6%) were overweight, 122 (7.6%) had normal BMI and became obese, and 63 (3.9%) progressed from overweight to obesity.

Associations between BMI trajectories groups and prostate cancer, overall and stratified by cancer aggressiveness, are presented in Table 2. We observed an increased risk of aggressive prostate cancer for men belonging to the “Overweight to obesity” trajectory. No association were observed for overall prostate cancer or low and intermediate aggressive prostate cancer.

**TABLE 2:** Associations between body mass index (BMI) trajectories and prostate cancer risk

BMI trajectories	Controls	Cases					
	n = 829 (%)	All		Low and intermediate^a^		Aggressive^b^	
		n = 781 (%)	OR [95% CI]^c^	n = 593 (%)	OR [95% CI]^c^	n = 176 (%)	OR [95% CI]^c^
Stable normal BMI	302 (36.5)	282 (36.1)	1.00 reference	218 (36.8)	1.00 reference	58 (33.0)	1.00 reference
Normal BMI to overweight	239 (28.8)	221 (28.3)	1.04 [0.80‐1.35]	171 (28.8)	1.03 [0.78‐1.37]	48 (27.3)	1.11 [0.71‐1.74]
Growing overweight	194 (23.4)	187 (23.9)	1.03 [0.78‐1.36]	137 (23.1)	0.96 [0.71‐1.30]	46 (26.1)	1.27 [0.80‐2.00]
Normal BMI to obesity	64 (7.7)	58 (7.4)	0.98 [0.64‐1.49]	46 (7.8)	1.05 [0.67‐1.63]	12 (6.8)	0.81 [0.36‐1.81]
Overweight to obesity	30 (3.6)	33 (4.2)	1.05 [0.60‐1.86]	21 (3.5)	0.81 [0.42‐1.54]	12 (6.8)	2.16 [1.00‐4.66]

^a^ Gleason ≤ 7 (3 + 4).

^b^ Gleason ≥ 7 (4 + 3).

^c^ ORs adjusted for age, family history of cancer at first degree, ethnicity.

Associations between BMI trajectories and prostate cancer, stratified on smoking status are presented in Table 3. Increased risks of aggressive prostate cancer were observed among never smokers for both “Normal BMI to overweight” and “Overweight to obesity” trajectories. A slight, but not significant, increased risk of aggressive prostate cancer was also observed among never smokers for men belonging to the “Normal BMI to obesity” trajectory. We did not find any association between BMI trajectories and prostate cancer risk among former or current smokers. Nevertheless, interaction between smoking status and BMI trajectories was not significant (*P* = .80).

**TABLE 3:** Associations between body mass index (BMI) trajectories and prostate cancer risk, stratified on smoking status

BMI trajectories	Controls	Cases					
	n = 829 (%)	All		Low and intermediate^a^		Aggressive^b^	
		n = 781 (%)	OR [95% CI]^c^	n = 593 (%)	OR [95% CI]^c^	n = 176 (%)	OR [95% CI]^c^
Never smokers							
Stable normal BMI	102 (44.0)	92 (40.2)	1.00 reference	77 (44.8)	1.00 reference	13 (26.5)	1.00 reference
Normal BMI to overweight	61 (26.3)	59 (25.8)	1.16 [0.71‐1.90]	40 (23.3)	0.91 [0.53‐1.58]	17 (34.7)	2.52 [1.07‐5.96]
Growing overweight	53 (22.8)	52 (22.7)	1.10 [0.66‐1.84]	37 (21.5)	0.92 [0.53‐1.62]	11 (22.5)	1.72 [0.68‐4.36]
Normal BMI to obesity	9 (3.9)	13 (5.7)	1.85 [0.70‐4.90]	11 (6.4)	1.74 [0.62‐4.84]	2 (4.1)	3.10 [0.56‐17.1]
Overweight to obesity	7 (3.0)	13 (5.7)	1.59 [0.57‐4.41]	7 (4.0)	0.82 [0.25‐2.69]	6 (12.2)	7.48 [2.05‐27.3]
Former smokers							
Stable normal BMI	137 (31.0)	148 (33.6)	1.00 reference	112 (33.1)	1.00 reference	33 (33.0)	1.00 reference
Normal BMI to overweight	137 (31.0)	138 (31.3)	0.99 [0.70‐1.42]	112 (33.1)	1.06 [0.73‐1.55]	26 (26.0)	0.87 [0.48‐1.59]
Growing overweight	106 (24.0)	103 (23.4)	0.95 [0.65‐1.38]	75 (22.2)	0.92 [0.61‐1.38]	28 (28.0)	1.17 [0.64‐2.13]
Normal BMI to obesity	44 (10.0)	37 (8.4)	0.74 [0.44‐1.26]	29 (8.6)	0.85 [0.49‐1.48]	8 (8.0)	0.44 [0.15‐1.35]
Overweight to obesity	18 (4.0)	15 (3.4)	0.74 [0.33‐1.65]	10 (3.0)	0.64 [0.36‐1.58]	5 (5.0)	1.19 [0.37‐3.90]
Current smokers							
Stable normal BMI	63 (40.7)	42 (38.2)	1.00 reference	29 (35.4)	1.00 reference	12 (44.5)	1.00 reference
Normal BMI to overweight	41 (26.4)	24 (21.8)	0.86 [0.43‐1.74]	19 (23.2)	0.96 [0.44‐2.09]	5 (18.5)	0.66 [0.20‐2.14]
Growing overweight	35 (22.6)	31 (28.2)	1.11 [0.54‐2.25]	24 (29.3)	1.19 [0.54‐2.60]	7 (25.9)	0.96 [0.31‐2.95]
Normal BMI to obesity	11 (7.1)	8 (7.3)	1.28 [0.42‐3.92]	6 (7.3)	1.36 [0.39‐4.74]	2 (7.4)	1.13 [0.21‐6.05]
Overweight to obesity	5 (3.2)	5 (4.5)	1.04 [0.24‐4.42]	4 (4.9)	1.10 [0.23‐5.40]	1 (3.7)	0.93 [0.10‐8.99]

^a^ Gleason ≤ 7 (3 + 4).

^b^ Gleason ≥ 7 (4 + 3).

^c^ ORs adjusted for age, family history of cancer at first degree, ethnicity.

In sensitivity analyses, results remained unchanged when analyses were restricted to men who had complete data on BMI.

## 4 DISCUSSION

In our study, based on an average of 5 BMI time‐points, we identified five BMI trajectories throughout adulthood. We did not find any association between BMI trajectories and prostate cancer overall. However, overweight men at age 20 who became obese in adulthood were at increased risk of aggressive prostate cancer. Moreover we showed that BMI trajectories resulting in overweight or obesity were more strongly associated with aggressive prostate cancer among never smokers.

To go further in the understanding of the relation between obesity and cancer risk, it has been suggested that BMI trajectory modeling may provide a more appropriate method to study the role of obesity in cancer risk compared to static measures of BMI.^10^

To the best of our knowledge, only four studies have explored the association between body shapes^11^ or BMI trajectories^12^, ^13^, ^14^ and prostate cancer incidence or mortality. One study, based on body shapes trajectories, did not find any association with advanced prostate cancer,^11^ while the three studies based on BMI trajectories observed a higher risk of overall^13^ or aggressive/fatal prostate cancer^12^, ^13^, ^14^ for men progressing from normal weight to overweight or obesity, compared to men with stable normal BMI, suggesting a role of life course obesity in prostate carcinogenesis.

There are many biological, metabolic and inflammatory mechanisms through which obesity may affect prostate carcinogenesis. Obesity is associated with lower levels of androgens^25^, ^26^ and there is some evidence that lower concentrations of testosterone result in the growth of more aggressive tumors.^27^ Obese men also have higher levels of insulin and insulin‐like growth factor (IGF‐I)^28^ and hyperinsulinemia has been shown to promote prostate cancer.^28^, ^29^, ^30^ Furthermore, obesity is associated with low grade chronic inflammation which may play a role in prostate cancer occurrence.^31^, ^32^

Besides, it is thought that the influence of early exposure to the etiology of prostate cancer is important,^33^ since carcinogenic processes were observed in prostatic tissue in 20‐year‐old men.^34^ Further studies are needed to confirm whether the timing and duration of obesity affect the development and progression of prostate cancer due to the complexity of the effects of obesity on carcinogenesis.

We stratified our analyses on smoking status based on the hypothesis that smoking could mitigate the association between obesity and cancer. Our findings that BMI trajectories resulting in overweight or obesity were more strongly associated with aggressive prostate cancer among never smokers, despite small numbers of participants after stratification, have also been reported by one study^14^ out of the two studies that stratifies on smoking status.^11^, ^14^

The biological mechanisms underlying the relationship between smoking and obesity are complex. Nicotine in cigarette increases energy expenditure and reduces appetite.^35^, ^36^ This may explain why smokers tend to have lower body weight than nonsmokers and why there is often weight gain related to smoking cessation.^37^, ^38^

It has been suggested that stratification on smoking status rather than adjustment may avoid potential residual confounding.^39^, ^40^ From a public health point a view, it is necessary to study separately smokers and nonsmokers. For nonsmokers, it is encouraged to maintain a body mass index in the normal range to prevent chronic diseases such as cancer. For smokers, a modest weight gain after quitting smoking may be tolerated as the benefits from smoking cessation would have positive impact on health.^40^

Our findings are based on a large population‐based case‐control study specifically designed to assess environmental and genetic factors in prostate cancer occurrence.^15^ The EPICAP study was carried out in the département of Hérault, France, which benefit from a cancer registry on which we were able to rely for the recruitment of cases. Overall, we were able to identify 1098 eligible cases, which was similar to what was expected according to prostate cancer cases that have been registered by the cancer registry in 2011, minimizing the potential for selection bias.^7^ To reflect the age distribution of cases, controls were randomly selected from the general population of the *département* of Hérault using quotas for age (5‐year age groups). To provide a control group similar to the male general population of the same age with respect to SES, quotas have also been established by SES, avoiding the ability for selection bias.^15^ The comparison of the SES distribution between our control group and the male general population of the *département* of Hérault after the selection process has shown no significant difference, indicating that the SES did not undergo significant selection bias.

We also compared the prevalence of overweight and obesity between the control group and the male general population and results were similar, suggesting that our control group is representative of the male general population of the same age.^41^

The same clinical research nurses collected data for cases and controls under the same conditions using a standardized questionnaire in order to reduce a possible differential classification bias that can persist in case‐control studies. Our results remained unchanged after adjustment for potential major confounding factors such as physical activity and smoking status, thus limiting potential confounding.

This analysis is based on the self‐declaration of weight, and is likely to lead to memory and declaration biases. A review of studies compared self‐reported and measured weight and showed that weight tends to be slightly underestimated by the participants.^42^ This tendency to underestimate has been confirmed in the National Nutrition and Health Survey (ENNS) that also showed greater difference with increased BMI.^43^ However, this underestimation is thought to affect both cases and controls. In addition, participants were asked to recall their weight every decade since the age of 20. In a study comparing self‐reported weight with actual measures taken at age 18, 30, and 40, a strong correlation between self‐reported and measured weights was observed (*r* = 0.87‐0.95) and recalls of weight were not significantly influenced by the passage of time.^44^ Nevertheless, we can reasonably assume that these errors might lead to non‐differential classification bias.

In conclusion, our results suggest that BMI trajectories resulting in overweight or obesity during adulthood are associated with an increased risk of prostate cancer. Furthermore, our results highlight the role of weight gain during adulthood on cancer risk. The association between obesity and prostate cancer is notably pertinent due to the large numbers of men affected by both diseases. The assessment of life course BMI may help identify men who are at increased risk of prostate cancer and may provide new prevention strategies.

## CONFLICT OF INTEREST

Nothing to declare.

## AUTHORS’ CONTRIBUTIONS

FM, PJL, XR, and BT contributed to study concept and design. FM, PJL, XR, and BT contributed to data acquisition. SC contributed to data management. CL, ECD, and FA contributed to statistical analysis. CL, ECD, FA, and FM contributed to analysis and interpretation of data. CL, ECD, and FM contributed to drafting of the manuscript.

## ETHICAL STATEMENT

The EPICAP study was approved by the review board of the French national institute of health and medical research (INSERM, n°01‐040, November 2010) and authorized by the French data protection authority (CNIL no 910485, April 2011).

## Data Availability

The data that support the findings of this study are available from the corresponding author upon reasonable request.
